# Altered Expression of Two-Pore Domain Potassium (K_2P_) Channels in Cancer

**DOI:** 10.1371/journal.pone.0074589

**Published:** 2013-10-07

**Authors:** Sarah Williams, Andrew Bateman, Ita O'Kelly

**Affiliations:** 1 Human Development and Health, Centre for Human Development, Stem Cells and Regeneration, Faculty of Medicine, University of Southampton, Southampton, United Kingdom; 2 Cancer Sciences, Faculty of Medicine, University of Southampton, Southampton, United Kingdom; University of Muenster, Germany

## Abstract

Potassium channels have become a focus in cancer biology as they play roles in cell behaviours associated with cancer progression, including proliferation, migration and apoptosis. Two-pore domain (K_2P_) potassium channels are background channels which enable the leak of potassium ions from cells. As these channels are open at rest they have a profound effect on cellular membrane potential and subsequently the electrical activity and behaviour of cells in which they are expressed. The K_2P_ family of channels has 15 mammalian members and already 4 members of this family (K_2P_2.1, K_2P_3.1, K_2P_9.1, K_2P_5.1) have been implicated in cancer. Here we examine the expression of all 15 members of the K_2P_ family of channels in a range of cancer types. This was achieved using the online cancer microarray database, Oncomine (www.oncomine.org). Each gene was examined across 20 cancer types, comparing mRNA expression in cancer to normal tissue. This analysis revealed all but 3 K_2P_ family members (K_2P_4.1, K_2P_16.1, K_2P_18.1) show altered expression in cancer. Overexpression of K_2P_ channels was observed in a range of cancers including breast, leukaemia and lung while more cancers (brain, colorectal, gastrointestinal, kidney, lung, melanoma, oesophageal) showed underexpression of one or more channels. K_2P_1.1, K_2P_3.1, K_2P_12.1, were overexpressed in a range of cancers. While K_2P_1.1, K_2P_3.1, K_2P_5.1, K_2P_6.1, K_2P_7.1 and K_2P_10.1 showed significant underexpression across the cancer types examined. This analysis supports the view that specific K_2P_ channels may play a role in cancer biology. Their altered expression together with their ability to impact the function of other ion channels and their sensitivity to environmental stimuli (pO2, pH, glucose, stretch) makes understanding the role these channels play in cancer of key importance.

## Introduction

Traditionally, the study of ion channels has focused on their roles in excitatory cells (neuronal, cardiac and secretory), however more recently, ion channels have been recognised for their roles in the behaviours of cancer cells and the development and progression of cancer. In the last 15 years increasing evidence supports the role of ion channels in mitogenesis, the control of cellular proliferation and apoptosis as well as cell migration and metastasis [1]–[8]. Overexpression of some ion channels has been linked to poor prognosis [9] while other channels are now recognised as potential biomarkers for particular cancer types [10], [11]. These reports, together with the potential of targeting ion channel function through pharmacological modulation, make understanding the role of ion channels in cancer biology of key importance.

K^+^ channels play fundamental roles in cell behaviours linked to cancer progression, including regulation of cell proliferation, migration, apoptosis and angiogenesis [2], [12]–[14]. Cell membrane potential (driven by K^+^ channel activity) plays an important regulatory role in cell cycle progression and proliferation, with highly proliferating cells displaying a more positive membrane potential than quiescent cells, while a transient membrane hyperpolarisation enables G1 progression [15]–[18]. The precise regulatory mechanisms are unclear but evidence supports two hypotheses. The first proposes that changes in membrane potential due to K^+^ channel activity modulates voltage-gated Ca^2+^ channels, thus impacting Ca^2+^ influx and downstream signalling [17], [19]. The alternative hypothesis proposes that the changes in cell volume seen during proliferation (cell swelling) and apoptosis (cell shrinkage) may be regulated by K^+^ channel activity [18], [20], [21]. In a similar manner, K^+^ channel control of membrane potential has been shown to impact cell migration through regulation of cell volume, pH and intracellular Ca^2+^ concentration. A direct impact of alteration in membrane potential on cytoskeletal polymerisation has also been demonstrated [14], [22], [23].

Altered K^+^ channel expression and/or function occurs in a range of cancer types, with ion channels from each of the K^+^ channel families (voltage sensitive (K_V_); calcium sensitive (K_Ca_); inwardly rectifying (K_ir_); and two-pore domain (K_2P_) channels) implicated in cancer development and progression. Within the K_V_ family, K_V_11.1 (hERG) shows altered expression in an array of cancer types and has been shown to impact cellular proliferation (melanoma, colorectal cancer and Barrett's esophagus), migration (melanoma, thyroid and breast cancer), malignant transformation (head & neck carcinoma) and apoptosis (gastric cancer). While K_V_11.1 is most frequently reported for its role in cancer, an array of other K^+^ channels have also been proposed as molecular components promoting cancer development and progression [9], [10], [24]–[80] (summarised in Table 1).

**Table 1:** Summary of potassium channel expression in cancer.

Channel	Expression detected	Behavioural impact	Ref
K_V_1.3	Breast, lung, lymphoma, pancreatic, prostate	Apoptosis, poor prognosis, proliferation	[24]–[28]
K_V_1.4	Gastrointestinal	Gene silencing	[29]
K_V_1.5	Brain	Increased survival	[30]
K_V_3.4	Head and neck	Proliferation	[31]
K_V_4.1	Breast, gastrointestinal	Proliferation	[32], [33]
K_V_10.1	Bone, breast, cervical, colorectal, esophageal, head and neck, kidney, leukemia (acute myeloid), ovarian	Biomarker, migration, proliferation, poor prognosis	[10], [34]–[42]
K_V_10.2	Brain, kidney	Proliferation	[42], [43]
K_V_11.1	Breast, colorectal, esophageal, gastrointestinal, head and neck, kidney, leukemia (acute myeloid), lung, melanoma, ovarian, retinoblastoma, thyroid	Migration, proliferation, poor prognosis,	[22], [34], [36], [42], [44]–[49]
K_Ca_1.1	Bone, brain, breast, ovarian, prostate	Apoptosis, metastases, microenvironment regulation, migration, proliferation	[9], [50]–[55]
K_Ca_2.3	Breast, colon, melanoma	Migration	[56]–[58]
K_Ca_3.1	Brain, breast, colorectal, melanoma, prostate	Migration, proliferation	[59]–[63]
K_ir_2.2	Breast, gastrointestinal, prostate	Cell cycle	[64]
K_ir_3.1	Breast, lung, pancreatic	Metastases, proliferation	[27], [65], [66]
K_ir_3.4	Aldosterone-producing adenomas	Mutations detected	[67]
Kir4.1	Brain	Migration, poor prognosis	[68], [69]
K_ir_6.1/K_ir_6.2	Brain, breast, melanoma, uterine	Apoptosis, cell cycle, proliferation	[70]–[73]
K_2P_2.1	Prostate	Proliferation	[74]
K_2P_3.1	Aldosterone-producing adenomas	Aldosterone production	[75]
K_2P_5.1	Breast	Proliferation	[76]
K_2P_9.1	Breast, colorectal, lung, melanoma	Apoptosis, migration, mitochondrial function, proliferation	[77]–[80]

Potassium channels identified in specific cancer types together with the predominant behavioural characteristics. Channels are divided into family groups, voltage-gated (K_V_), calcium-gated (K_Ca_), inward rectifying (K_ir_) and two-pore domain (K_2P_).

The potential role of K_2P_ channels in cancer is of particular interest. These channels conduct outward K^+^ background currents and are active at resting membrane potentials, thus they have a direct influence on baseline cellular activity of cells at rest including membrane potential, calcium homeostasis and cell volume regulation. K_2P_ channels also show sensitivity to physiological stimuli including pH, oxygen tension, glucose concentration and stretch; key physiological parameters which are disrupted within the cancer cells and their environment [81]–[83].

Of the 15 mammalian K_2P_ family members, four K_2P_ channels (K_2P_2.1 (TREK-1), K_2P_3.1 (TASK-1), K_2P_9.1 (TASK-3) and K_2P_5.1 (TASK-2)) have already been implicated in cancer. In 2003, Mu *et al*. [77] described KCNK9, the gene encoding K_2P_9.1, as a potential proto-oncogene where genomic overexpression of the gene was detected in 10% of breast carcinomas and the protein was detected in 44% of breast tumours by immunohistochemistry but not in normal tissue controls. The oncogenic ability (measured by proliferative advantage) was demonstrated to depend upon a functional channel [84]. K_2P_9.1 immunopositivity has subsequently been reported in colorectal carcinomas [78] and melanoma tissue samples [85].

Increased K_2P_2.1 expression was detected in prostate adenocarcinoma samples compared to normal prostate epithelium and reduced proliferation of prostate cancer cell lines was observed when K_2P_2.1 was experimentally knocked down [74].

A study by Nogueira *et al*. (2010) [75] linked K_2P_3.1 expression to aldosterone production in both aldosterone-producing adenomas and normal adrenals, and proposed K_2P_3.1 may play a role in Ca^2+^ signalling regulation. Equally, K_2P_3.1 and K_2P_9.1 have previously been reported to play a role in K^+^-dependent apoptosis in granule cell neurons in culture [86].

Transcriptome analysis in human ductal breast epithelial tumour cell line, T47D, following either stimulation with either estrogen receptor (ER) α which induces proliferation or ERβ which has antiproliferative effects showed that K_2P_5.1 mRNA was upregulated by ERα signalling [87]. mRNA, protein and functional expression (acid-sensitive outward currents) of K_2P_5.1 was reported to increase in response to 17β-estradiol stimulation of ERα signalling in T47D and human breast adenocarcinoma cell line, MCF-7. While experimental knockdown of K_2P_5.1 moderately reduced basal proliferation of T47D cells, a significantly greater reduction in estrogen-induced proliferation was observed [76].

Evidence from these studies supports the hypothesis that alterations to the expression or function of K_2P_ channels in cancer cells may play a role in cancer development and progression. Targeting these channels may lead to novel cancer therapies; we therefore sought to determine the transcript expression of each of the K_2P_ channels in a range of cancers using an online cancer microarray database, Oncomine (www.oncomine.org, Compendia biosciences, Ann Arbor, MI, USA). This information documents changes in the expression of the K_2P_ family members in a range of cancer types and provides a valuable resource to enable further investigation into the protein expression and potential roles of these important channels in cancer progression.

## Methods

Analysis of KCNK mRNA expression in cancer tissue samples (meta-analysis of KCNK genes and related statistical analyses) were performed using the online cancer microarray database, Oncomine (www.oncomine.org, Compendia biosciences, Ann Arbor, MI, USA). Oncomine collects publicly available cancer microarray data and processes all data imposing the same criteria [88]. The mRNA expression data is organised into cancer types defined within the original publications. mRNA expression data was extracted from Oncomine between August 2012 and January 2013. Citations for all primary studies used together with information on cancer type and staging (where available) is provided in Table S1 in File S1.

Only datasets examining KCNK gene mRNA expression in cancer tissue which was matched with normal tissue controls (cancer vs. normal) were included in this study. Threshold criteria had to be achieved by each study for inclusion in the analysis. The threshold search criteria used for this study were a p-value<0.05, a fold change >2 and a gene rank percentile <10%. P-values presented in this study for differential expression analysis of KCNK genes were calculate by Oncomine using a two-sided Student's t-test and multiple testing correction [86], [87]. Multiple testing correction was performed using the false discovery rate method, where corrected p-values (Q-values) were calculated as Q = NP/R (where P = p-value, N = total number of genes and R is the sorted rank of p-value) [88], [89]. In this study a p-value less than 0.05 was considered significant. Fold change is defined as the linear change in mRNA for the gene of interest in cancer tissue when compared to the normal expression level for that tissue, in this case a fold change of 2 and greater was included for analysis. For each dataset the genes studied are ranked by their p-value. The gene rank percentile is the percentage ranking of the gene of interest compared to all other genes analysed in that dataset based on p-values. The average number of genes examined in the microarray data presented in this study was approximately 14,000 genes. Datasets in which the gene of interest was in the top 10% of genes changed were included. These threshold values are connected by the Boolean AND, therefore an analysis was only classed as above threshold when it met all three criteria.

Initially KCNK genes (KCNK1–18) were examined across a range of 20 cancer types, which have been grouped by their tissue of origin (Table S2 in File S1), comparing mRNA expression in that cancer type to normal tissue controls. Gene summary view in Oncomine was utilised during this analysis and presented here with expression ranking indicated by colour shading. Expression colouring for a gene in a particular cancer relates to the gene rank percentile for the highest ranking above threshold analysis.

Further analysis was performed on each KCNK gene, for expression in the most prevalent cancer types based on GLOBACON 2008 WHO rankings (http://globocan.iarc.fr/) [90]. Lymphoma, myeloma, sarcoma, liver and ovarian cancers were removed from further analysis due to low KCNK expression. The subtype ‘other cancers’ which is defined as cancers which do not fall into the prescribed subtypes (e.g. uterine and adrenal cancers) was also removed from further analysis as the large diversity of cancer subtypes within this group would make detailed analysis uninformative. Using the threshold criteria described previously all above threshold analyses for each KCNK gene was extracted from Oncomine and complied.

Once all above threshold data for each KCNK gene had been complied, comparative meta-analysis was performed on cancer subtype with more than five datasets (n≥5) available, this analysis provided a median gene rank and median p-value for that cancer subtype.

## Results and Discussion

### KCNK genes show altered expression across different cancers

**Figure 1:**
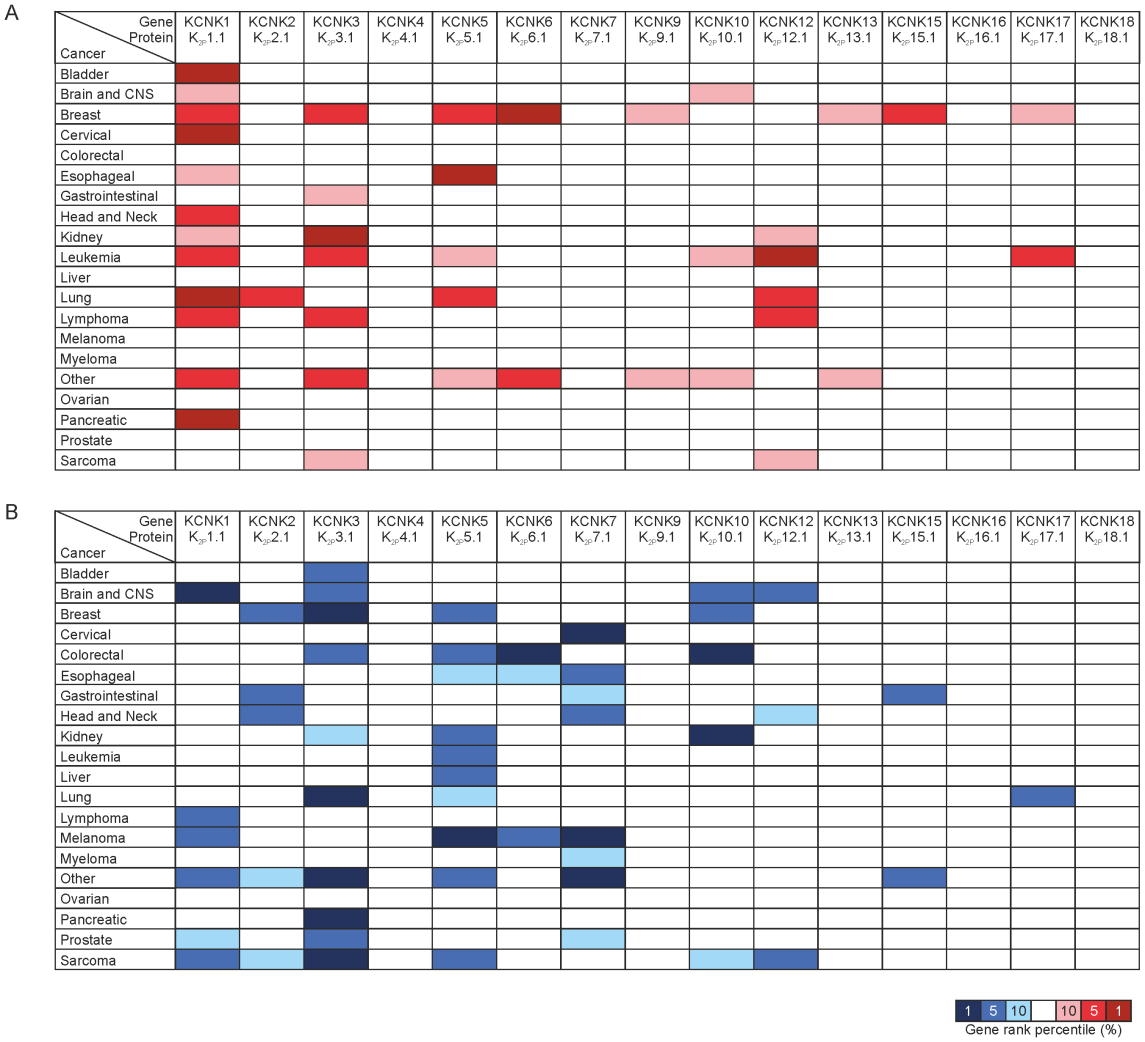
Expression of KCNK genes across different cancers. Expression of KCNK genes (KCNK1–18) in 20 cancers compared to normal tissue controls. Shown is the gene and protein names for each channel. A) overexpression of KCNK genes. B) underexpression of KCNK genes. Cancer types are organised by their tissue of origin, the degree of colour correlates to the gene rank percentile of the highest ranking analyses. Search criteria were for mRNA datasets and cancer vs. normal analysis only, with threshold values of p-value<0.05, fold change >2 and gene rank percentile <10%.

KCNK genes 1–18 (with the omission of KCNK8, KCNK14 and KCNK11 which were ascribed proteins but subsequently withdrawn due to nomenclature duplication) encode the mammalian family of K_2P_ channels [91]. Initially to obtain a global view of changes in K_2P_ channel expression in cancer, we used the Oncomine cancer microarray database to analyse the alterations observed in KCNK gene mRNA expression in the 20 most commonly diagnosed cancers, grouped by their tissue of origin, compared to normal tissue controls. For inclusion in the analysis, changes in gene expression compared to normal controls had to fulfil threshold criteria of achieving a p-value<0.05, a fold change >2 and a gene rank percentile <10%. The gene rank percentile values for each of the 15 KCNK genes in cancers compared to normal tissue controls were examined and the percentile of the highest ranking analyses are shown for each KCNK gene and each cancer tissue type in Figure 1. Performing analysis in this way enabled comparison of alterations in gene expression to be performed between different microarray experiments and revealed that all KCNK genes with the exception of KCNK4 (K_2P_4.1 or TRAAK), KCNK16 (K_2P_16.1 or TALK1) and KCNK18 (K_2P_18.1 or TRESK) show altered expression in the 20 cancer types examined when compared to normal tissue controls (Figure 1A & B). Cancers from fourteen tissue types showed over-expression of more than one KCNK gene (Figure 1A) with five cancer tissue types (breast, kidney, leukaemia, lung, lymphoma) showing over-expression of three or more KCNK genes (Figure 1A). While broad cancer tissue types are considered in this initial analysis and include a range of different cancer diseases, they provide valuable preliminary information on the expression of KCNK genes in cancer and further analysis taking into account specific cancer subtypes (e.g. acute versus chronic leukaemia) was performed for specific channels in subsequent analyses.

When examining underexpression of KCNK genes, cancer from 19 of the 20 tissue types analysed showed decreased expression of one or more KCNK genes when compared to normal tissue expression (Figure 1B). Six K_2P_ family members (KCNK1, KCNK2, KCNK3, KCNK5, KCNK7 and KCNK10) show underexpression in over 5 different cancer tissue types of (Figure 1B). While 10 different cancer tissue types (brain, breast, colorectal, gastrointestinal, head and neck, kidney, lung, melanoma, prostate and sarcoma) show underexpression of at least three KCNK genes (Figure 1B). Strikingly, specific K_2P_ channels show increased mRNA expression in some cancer tissues while decreased expression in others. This is particularly apparent for KCNK1, KCNK3, KCNK5 and KCNK6, which displayed mRNA expression changes (either up or down in distinct cancers) which rank them in the top 1% of genes showing altered expression for those cancers. KCNK1, for example, is in the top 1% of genes showing overexpression in bladder, cervical, lung and pancreatic cancers, while in cancers of the central nervous system KCNK1 shows one of the highest reductions in expression when compared to normal tissue controls (Table 2). These analyses suggest that the impact of down-regulation of K_2P_ channels on cell function may be an equally important alteration as increased expression in cancer biology.

**Table 2:** TWIK family members expression in cancer.

Gene	Cancer	Subtype	Above threshold analyses					Median values		
				p-value	Fold change	%	Ref	p-value	Gene rank	n
KCNK1	Brain	Glioblastoma	↓	1.72E-24	−9.574	2	[9]	5.14E-06	560.5	8
			↓	1.80E-14	−20.541	3	[10]			
			↓	1.07E-08	−8.483	3	[3]			
			↓	1.03E-05	−13.309	4	[6]			
			↑	5.89E-04	3.178	6	[5]			
	Breast	Ductal	↑	5.23E-04	2.515	5	[16]	0.002	2547	12
			↑	7.18E-04	3.965	5	[12]			
			↑	1.00E-03	2.405	5	[15]			
			↑	4.00E-03	2.661	9	[12]			
			↑	1.10E-02	2.4	9	[13]			
		Lobular	↑	2.20E-02	2.177	4	[13]	0.031	3848	5
	Cervical	Squamous Cell	↑	9.70E-13	2.949	1	[20]	0.043	3326	5
	Leukaemia	Acute Lymphocytic	↑	4.00E-03	2.173	5	[42]	9.04E-07	4799	7
	Lung	Adenocarcinoma	↑	3.59E-07	3.984	2	[52]	8.51E-13	511	7
			↑	5.38E-07	2.141	3	[46]			
			↑	2.35E-05	4.641	1	[47]			
			↑	6.21E-05	2.137	8	[51]			
		Squamous cell	↑	5.98E-08	2.138	9	[49]	0.002	852	6
			↑	2.61E-06	7.79	2	[47]			
	Pancreas	Adenocarcinoma	↑	9.83E-10	3.526	5	[71]	0.008	787.5	8
			↑	2.61E-04	6.584	3	[72]			
			↑	1.41E-04	6.62	5	[73]			
			↑	1.21E-08	4.613	1	[74]			
			↑	2.00E-03	2.685	9	[75]			
KCNK6	Breast	Ductal	↑	2.77E-19	2.161	9	[17]	0.076	5236	10
			↑	1.00E-03	2.765	1	[14]			
	Colorectal	Adenocarcinoma	↓	1.77E-18	−2.071	4	[26]	0.028	4860	11
			↓	2.38E-15	−2.11	7	[26]			
			↓	9.37E-15	−2.136	1	[26]			
KCNK7	Cervical	Squamous cell	↓	5.62E-10	−6.76	1	[21]	7.99E-04	519	5
			↓	1.86E-08	−3.055	1	[22]			
			↓	7.99E-04	−3.315	5	[22]			
	Gastrointestinal	Adenocarcinoma	↓	1.60E-02	−2.336	10	[29]	0.446	9583	5

The above threshold data for TWIK family members; KCNK1, KCNK6 and KCNK7 is shown. Data is divided into each cancer type and subtypes within that cancer. The p-value, fold change and gene rank percentile (%) for data which scored above threshold values (p-value<0.05, fold change >2 and gene rank percentile <10%) are shown. Comparative meta-analysis was performed using all available analyses for a given cancer subtype which provides median gene rank and median p-value. Overexpression ↑ and underexpression ↓ are indicated.

### KCNK expression in specific cancer types

The 15 members of the K_2P_ channel family are divided into 6 separate groupings on the basis of their sequence homology and defining biophysical characteristics. The expression of each gene in each of the 14 cancer tissue types (6 tissues were excluded from this analysis due to low dataset numbers or high cancer subtype diversity) was studied in detail using the analysis threshold values as before (p-value<0.05, fold change >2 and gene rank percentile <10%) and the results are presented for each channel group (Tables 2, 3, 4, 5, 6 & Table S3 in File S1). Data from comparative meta-analysis performed for specific KCNK genes in cancer sub-types in which a sufficient number of microarray studies (n≥5) examining these genes were available are presented in Tables 2, 3, 4, 5, 6 and was performed using all datasets in which the gene of interest was examined and not just those which ranked above threshold values. Meta-analysis provided the median gene rank and median p-value, thus enabling comparison across different microarray studies. If the median ranked analysis had a significant p-value it indicated that the expression trend for that gene was likely to be altered in that cancer subtype. If less than 5 independent studies for any of the genes in a particular cancer subtype were not available on Oncomine, meta-analysis of data which reached threshold was not performed but instead was collated and presented in Table S3 in File S1.

**Table 3:** TREK family members expression in cancer.

Gene	Cancer	Subtype	Above threshold analyses					Median value		
				p-value	Fold change	%	Ref	p-value	Gene rank	n
KCNK2	Breast	Invasive	↓	5.70E-05	−2.23	4	[19]	0.349	8095	11
	Lung	Squamous cell	↑	2.98E-04	2.111	5	[48]	0.696	6505	5
KCNK10	Brain	Glioblastoma	↓	1.81E-17	−4.843	5	[9]	5.03E-05	908	5
			↓	1.56E-10	−2.974	6	[10]			
			↑	8.63E-04	2.547	7	[5]			
	Breast	Ductal	↓	1.00E-03	−2.294	2	[18]	0.15	6686.5	10
			↓	3.85E-04	−3.523	2	[14]			
	Colorectal	Adenocarcinoma	↓	1.74E-25	−7.227	1	[26]	8.12E-07	372.5	14
			↓	3.19E-22	−7.914	2	[26]			
			↓	2.85E-18	−6.275	1	[26]			
			↓	2.07E-14	−4.83	2	[26]			
			↓	1.11E-07	−6.275	2	[26]			
			↓	3.42E-07	−2.503	3	[26]			

The above threshold data for TREK family members; KCNK2 and KCNK10 is shown. Data is divided into each cancer type and subtypes within that cancer. The p-value, fold change and gene rank percentile (%) for data which scored above threshold values (p-value<0.05, fold change >2 and gene rank percentile <10%) are shown. Comparative meta-analysis was performed using all available analyses for a given cancer subtype which provides median gene rank and median p-value. Overexpression ↑ and underexpression ↓ are indicated.

**Table 4:** TASK family members expression in cancer.

Gene	Cancer	Subtype	Above threshold analyses					Median value		
				p-value	Fold change	%	Ref	p-value	Gene rank	n
KCNK3	Brain	Glioblastoma	↓	6.20E-08	−5.468	10	[10]	0.007	1486	7
			↓	2.61E-05	−4.471	3	[10]			
	Breast	Invasive	↑	4.41E-17	2.782	4	[11]	0.005	8863	13
			↑	1.50E-02	2.958	6	[14]			
			↓	1.00E-03	−2.375	7	[17]			
	Colorectal	Adenoma	↓	2.37E-04	−2.493	10	[24]	2.37E-04	1814	5
			↓	2.00E-03	−4.175	5	[24]			
	Gastrointestinal	Adenocarcinoma	↑	2.85E-04	3.567	6	[28]	1	10604	6
	Kidney	Clear cell	↑	1.53E-14	8.407	1	[36]	1.14E-04	990	6
			↑	2.57E-07	6.014	5	[36]			
			↑	4.01E-05	4.541	7	[38]			
			↑	1.89E-04	6.344	6	[41]			
	Leukemia	Acute lymphocytic	↑	1.30E-02	2.177	9	[42]	0.994	8503	7
	Lung	Adenocarcinoma	↓	6.55E-34	−4.136	1	[50]	4.33E-11	146.5	6
			↓	8.44E-20	−6.89	1	[49]			
			↓	8.67E-11	−7.375	2	[52]			
			↓	4.11E-10	−2.367	1	[51]			
			↓	2.54E-06	−7.399	3	[47]			
			↓	1.08E-04	−3.803	4	[48]			
		Squamous cell	↓	5.90E-20	−12.756	2	[49]	5.90E-20	343	5
			↓	3.86E-06	−8.471	3	[47]			
			↓	1.58E-05	−4.28	3	[48]			
			↓	2.00E-03	−2.422	7	[53]			
	Pancreas	Adenocarcinoma	↓	7.34E-06	−6.459	1	[74]	2.46E-07	2997	7
			↓	4.72E-05	−5.03	1	[72]			
			↓	1.19E-04	−2.191	3	[75]			
	Prostate	Carcinoma	↓	2.72E-08	−2.034	3	[77]	0.029	1515	13
			↓	1.02E-04	−2.638	2	[79]			
			↓	8.94E-04	−3.106	4	[76]			
KCNK9	Breast	Invasive	↑	1.16E-12	3.95	9	[11]	0.459	10188.5	14
KCNK15	Breast	Ductal	↑	1.00E-03	5.046	6	[12]	0.008	1578	6
			↑	8.00E-03	2.283	9	[12]			
			↑	4.10E-02	8.774	8	[14]			
	Gastrointestinal	Adenocarcinoma	↓	3.00E-03	−2.189	5	[29]	0.043	2990	5

The above threshold data for TASK family members; KCNK3, KCNK9 and KCNK15 is shown. Data is divided into each cancer type and subtypes within that cancer. The p-value, fold change and gene rank percentile (%) for data which scored above threshold values (p-value<0.05, fold change >2 and gene rank percentile <10%) are shown. Comparative meta-analysis was performed using all available analyses for a given cancer subtype which provides median gene rank and median p-value. Overexpression ↑ and underexpression ↓ are indicated.

**Table 5:** TALK family members expression in cancer.

Gene	Cancer	Subtype	Above threshold analyses					Median value		
				p-value	Fold change	%	Ref	p-value	Gene rank	n
KCNK5	Breast	Ductal	↑	2.14E-04	2.977	3	[12]	0.233	4729	9
			↓	7.70E-04	−3.629	4	[12]			
			↓	2.00E-03	−2.498	2	[18]			
			↓	1.00E-03	−3.856	6	[12]			
	Colorectal	Adenocarcinoma	↓	2.42E-12	−3.18	3	[26]	2.35E-07	1052	11
			↓	1.95E-11	−2.498	5	[26]			
			↓	7.86E-08	−3.199	2	[26]			
KCNK17	Breast	Invasive	↑	2.20E-02	3.265	8	[14]	0.752	14529	12

The above threshold data for TALK family members; KCNK5 and KCNK17 is shown. Data is divided into each cancer type and subtypes within that cancer. The p-value, fold change and gene rank percentile (%) for data which scored above threshold values (p-value<0.05, fold change >2 and gene rank percentile <10%) are shown. Comparative meta-analysis was performed using all available analyses for a given cancer subtype which provides median gene rank and median p-value. Overexpression ↑ and underexpression ↓ are indicated.

**Table 6:** THIK family member, KCNK13 expression in cancer.

Gene	Cancer	Subtype	Above threshold analyses					Median value		
				p-value	Fold change	%	Ref	p-value	Gene rank	n
KCNK13	Breast	Invasive	↑	3.35E-12	3.193	10	[11]	0.399	10349	11
			↑	4.99E-08	2.05	10	[17]			

The above threshold data for THIK family member; KCNK13 is shown. Data is divided into each cancer type and subtypes within that cancer. The p-value, fold change and gene rank percentile (%) for data which scored above threshold values (p-value<0.05, fold change >2 and gene rank percentile <10%) are shown. Comparative meta-analysis was performed using all available analyses for a given cancer subtype which provides median gene rank and median p-value. Overexpression ↑ and underexpression ↓ are indicated.

### Two-pore domain weak inward rectifying K^+^ (TWIK) channel family

TWIK channels include KCNK1 (K_2P_1.1, TWIK1), KCNK6 (K_2P_6.1, TWIK2) and KCNK7 (K_2P_7.1). None of these channels have previously been implicated in playing a role in cancer, but analysis presented here reveals a significant overexpression of KCNK1 in the majority of cancers analysed (12 out of 20 cancer tissue types show overexpression with KCNK1 ranked in the top 10% of most altered genes) while 6 cancer tissue types showed KCNK1 underexpression when compared to normal tissue (Figure 1). KCNK6 was found to be among the top 1% of genes overexpressed in breast cancer and top 1% of genes underexpressed in colorectal cancer. While KCNK7 failed to show overexpression in any of the cancer types examined it showed significant underexpression in a range of cancers and was in the top 1% of underexpressed genes in both melanoma and cervical cancers (Figure 1).

Cancer subtypes in which KCNK1 showed above threshold changes in expression are presented in Table 2 (if sufficient studies were available for meta-analysis (n≥5)) or Table S3 in File S1 (if insufficient number of studies were available for meta-analysis (n≤4)). All cancer sub-types with KCNK1 overexpression eligible for meta-analysis were found to show significant levels of overexpression (median p-value≤0.05; Table 2). Lung adenocarcinomas had the most significant increase in expression compared to normal tissue, with a 3.22±0.64 mean fold increase from the 4 studies which reached threshold for inclusion and a median p-value of 8.51E-13 (n = 7; Table 2). While, pancreatic adenocarcinomas showed the highest mean (± SEM) fold increase (4.80±0.79) in KCNK1 transcript compared to the normal controls in the 5 studies above threshold criteria.

Brain cancers of glial cell origin (astrocytoma, glioblastoma, oligodendrioglioma), medulloblastoma and melanomas all showed significant down regulation of KCNK1 with respect to normal control tissues (Table S3 in File S1). All but glioblastoma had insufficiently high number of independent analyses to enable inclusion in comparative meta-analysis (Table S3 in File S1), while in glioblastoma 4 above threshold analyses showed underexpression ranging from 8 to 20 fold decreases in KCNK1 transcript expression whereas one study showed a 3 fold increase of KCNK1 mRNA (Table 2). Comparative meta-analysis of all 8 studies in which KCNK1 transcript expression was examined revealed an overall significant (p = 5.14E-6) decreased expression of KCNK1 in glioblastoma (Table 2). KCNK1 is not the only gene to show apparently conflicting expression profiles but this may be due to the broad groupings in each of the cancer types. Significantly this is also observed for KCNK10 in brain glioblastoma (Table 3).

While KCNK6 shows overexpression in both ductal (average fold change 2.46; n = 2) and invasive (fold change 3.57 (n = 1)) breast cancer, overall, KCNK6 and KCNK7 show more transcript underexpression (Table 2). Though, meta-analysis of KCNK6 expression in ductal breast cancer found the increased expression not to reach significance (p = 0.076; n = 10). Both KCNK6 and KCNK7 show underexpression in melanoma and oesophageal adenocarcinomas. KCNK6 showed significant decreased expression in colorectal adenocarcinoma (median p-value = 0.028; n = 11) with a mean (± SEM) fold decreased expression of 2.11±0.02 in the 3 above threshold analyses for underexpression. KCNK7 underexpressed in Barrett's oesophagus when compared to normal tissue controls but insufficient numbers of studies were available to enable further analysis (Table S3 in File S1). KCNK7 showed significant down-regulation in cervical squamous cell carcinoma (median p-value of 7.99E-04; n = 5) with a mean (± SEM) fold decreased expression of 4.37±1.19. A decreased expression of KCNK7 observed in gastrointestinal adenocarcinomas failed to show significance following meta-analysis (median p-value 0.446, n = 5) and achieved a median gene rank of 9583 out of circa 14000 genes suggesting that alterations in KCNK7 expression are less important in gastrointestinal adenocarcinomas.

### TWIK-related K^+^ (TREK) channel family

The TREK family has 3 family members KCNK2 (K_2P_2.1, TREK1), KCNK4 (K_2P_4.1, TRAAK) and KCNK10 (K_2P_10.1, TREK2). KCNK4 failed to show altered expression above the set thresholds in the 20 cancers examined and therefore was not further analysed.

KCNK2 was among the top 5% of genes over expressed in lung cancers and under expressed in breast, gastrointestinal and head and neck cancers (Figure 1). KCNK10 was among the top 1% of genes underexpressed (compared to normal tissue controls) in colorectal and kidney cancers while in breast and brain cancers KCNK10 was among the top 5% of genes underexpressed (Figure 1A & B). As seen with KCNK1 in glioblastoma, two of the above threshold analyses show decreased KCNK10 expression (compared to normal tissue controls) ranging from 2.9 to 4.8 fold decreases, while a third analysis shows a 2.5 fold increase in KCNK10 expression. Meta-analysis including all studies in which KCNK10 expression was examined in glioblastoma cancer revealed a significant decreased expression (median p-value = 5.03E-05; n = 5) but while clear changes in KCNK10 expression levels are observed in glioblastoma further studies and analysis are required to determine the nature of these alterations. KCNK10 was also ranked in the top 10% of over-expressed genes in acute myeloid leukemia (Figure 1A & Table S3 in File S1; n = 4) but insufficient studies were available to enable robust meta-analysis to be performed to determine the significance of this change. KCNK10 shows decreased expression in breast ductal and lobular carcinomas and colorectal adenoma, adenocarcinoma and carcinoma as well as kidney clear cell carcinoma (Table 3 & Table S3 in File S1). Only breast ductal carcinoma and colorectal adenocarcinoma had sufficient number of studies to enable meta-analysis (Table 3). This analysis revealed the changes in breast ductal carcinoma not to be significant (median p-value = 0.15; n = 5) while colorectal adenocarcinoma showed significant decreased expression of KCNK10 (median p-value = 8.12E-07; n = 14)

KCNK2 showed decreased expression in invasive breast cancer, gastrointestinal adenocarcinoma and head and neck squamous cell carcinoma but these studies either failed to be included in meta-analysis due to low study numbers or failed to show significance following meta-analysis (Table 3 & Table S3 in File S1).

These data while limited by the sample size provide sufficient evidence to warrant further investigation into the role of KCNK10 in both glioblastoma and colorectal adenocarcinoma.

### TWIK-related acid sensitive K^+^ (TASK) channel family

The TASK family has three members KCNK3 (K_2P_3.1, TASK1), KCNK9 (K_2P_9.1, TASK3) and KCNK15 (K_2P_15.1, TASK5).

KCNK3 showed altered expression in the majority of cancers examined (13 out of 20) and was in the top 1% of up-regulated genes in kidney cancer and top 5% of up-regulated genes in breast, leukaemia and lymphoma (Figure 1A). KCNK3 was in the top 1% of under-expressed genes in sarcoma, breast, lung and pancreatic cancers. KCNK3 was also in the top 5% of under-expressed genes in cancers of the CNS, bladder, colorectal and prostate (Figure 1B). Detailed meta-analysis of cancer subtypes with decreased KCNK3 expression revealed underexpression to be significant in pancreatic adenocarcinoma (median p-value = 2.46E-07; n = 7), lung adenocarcinoma (median p-value = 4.33E-11; n = 6), colorectal adenoma (median p-value = 2.37E-04; n = 5) and glioblastoma (median p-value = 0.007; n = 7; Table 4). Lung squamous cell carcinoma showed both the highest level of significance following meta-analysis of 5 studies in which KCNK3 gene expression was examined (median p-value = 5.90E-20) and highest mean fold decrease in KCNK3 expression from the 4 studies which reached threshold (6.98±2.30; Table 4).

Analysis of KCNK3 transcript expression in specific cancers within the broad cancer types shows significant increase in KCNK3 expression in invasive breast (median p-value = 0.005) and clear cell kidney (median p-value = 1.14E-04) cancers with a 4.5 to 8.4 fold increase in expression in clear cell kidney carcinomas when compared to normal tissue controls (Table 4).

While K_2P_9.1 has previously been identified in breast, colon and melanoma cancers [78], [82], [83], KCNK9 only showed an above threshold analysis for invasive breast carcinomas (p-value = 1.16E-12; Table 4). When comparative meta-analysis was performed with 14 analyses examining KCNK9 in invasive breast carcinomas, the changes were found not to be significant (median p-value = 0.459).

KCNK15 shows significant overexpression, by comparative analysis, in ductal breast carcinomas (median p-value = 0.008; 5.37±1.88 mean fold increase in 3 above threshold analyses) and underexpression in gastrointestinal adenocarcinomas (median p-value = 0.043; Table 4).

### TWIK-related alkaline pH activated K^+^ (TALK) channel family

The TALK family has three family members KCNK5 (K_2P_5.1, TASK2), KCNK16 (K_2P_16.1, TALK1) and KCNK17 (K_2P_17.1, TALK2). KCNK16 failed to show altered expression above the set thresholds in the 20 carcinomas examined initially and therefore was not further analysed.

KCNK5 showed altered expression in 50% of cancers examined. It was in the top 1% of up-regulated genes in esophageal cancers and top 5% of up-regulated genes in breast and lung cancers (Figure 1A). Decreased expression of KCNK5 was observed in a wider range of cancer subtypes with KCNK5 in the top 1% of under-expressed genes in melanoma and top 5% of under-expressed genes in breast, colorectal, kidney, leukaemia, liver cancers and sarcoma (Figure 1B). Although not all cancer subtypes which demonstrated changes in expression of KCNK5 had sufficient number of studies for comparative analysis (Table S3 in File S1), meta-analysis of colorectal adenocarcinoma studies showed a significant decrease in KCNK5 expression (median p-value = 2.35E-07; n = 11; Table 5) with a mean fold decrease of 2.96±0.23 (n = 4). Further studies are required to determine if the down-regulation of KCNK5 observed in other cancer subtypes are also significant.

A single study reached the threshold criteria and showed a 3.26 fold increase in KCNK17 expression in invasive breast carcinomas (Table 5). However when comparative meta-analysis was performed with all analyses examining KCNK17 in invasive breast carcinomas (n = 12) it was found not to be significant (median p-value = 0.752) suggesting the study which reached threshold may not be representative of KCNK17 expression in breast cancer.

### Two pore domain halothane inhibited K^+^ (THIK) channel family

The THIK family has two family members KCNK12 (K_2P_12.1, THIK1) and KCNK13 (K_2P_13.1, THIK2).

KCNK12 showed altered expression compared to normal tissue controls in 7 of the 20 cancer types examined with both overexpression and underexpression observed (Figure 1 & Table S3 in File S1). Above threshold reductions in KCNK12 expression were observed in astrocytoma and glioblastoma, while increased expression was seen in acute lymphocytic leukaemia and lung adenocarcinoma but insufficient sample sizes for any of these cancer subtypes prevented any comparative meta-analysis of KCNK12 to be performed. KCNK13 showed two above threshold analysis for invasive breast carcinomas with 2.62±0.57 mean (± SEM) fold increase in KCNK13 expression. However when comparative analysis was performed with 11 analyses examining KCNK13 in invasive breast carcinomas, altered expression of KCNK13 failed to reach significance (median p-value = 0.399; Table 6).

### Potential role for K_2P_ channels in cancer therapy

This study provides a comprehensive overview of the current data available on KCNK gene family expression in cancer and clearly demonstrates altered expression of these genes is observed in the majority of cancer types examined. In all of the 20 cancers examined with the exception of ovarian cancer KCNK genes were found in the top 10% of altered genes and were in the top 1% in 13 of these cancers. In several instances, specific cancer subtypes show changes in a number of KCNK genes. Specifically brain glioblastoma showed significant down regulation of KCNK1, KCNK3 and KCNK10; while KCNK12 also showed decreased expression but insufficient studies were available to enable comparative analysis. Likewise, breast ductal cancer showed significant increased expression of KCNK1, KCNK6 and KCNK15. Noteworthy is the observation that in some cancer subtypes overexpression of one KCNK gene occurs alongside underexpression of another, this is observed in lung adenocarcinoma, lung squamous and pancreatic adenocarcinomas, where in all three of these cancer subtypes KCNK1 shows significant over-expression while KCNK3 is significantly under-expressed. As specific K_2P_ family members show altered sensitivities to different modulators such as intracellular and extracellular pH (TWIK, TREK, TASK, TALK), hypoxia and reactive oxygen species (TASK, TALK, THIK) and glucose concentration (TASK), changing the relative expression of different K_2P_ channels may impact the response of cells to environmental cues [81]–[83], [91]–[96]. Moreover, either increased or decreased expression of these channels has the potential to induce membrane hyperpolarisation or depolarisation respectively. As noted previously, alterations to membrane potential is recognised to drive changes in cell proliferation, apoptosis and migration [14]–[18], [21]. As K_2P_ channels are active over physiological membrane potential ranges, this means these channels are ideally positioned to directly impact cellular membrane potential at rest. This, together with their acute sensitivity to the internal and external environment of the cell which is known to change in the cancer microenvironment means that altered expression of these channels may provide cancer cells with a survival advantage.

Understanding the molecular and pharmacological regulation of these channels together with a detailed knowledge of the expression of these channels in cancer will enable these important membrane proteins to be considered as potential therapeutic targets in cancer treatment.

## Supporting Information

File S1 **Contains Tables S1, S2, and S3. Oncomine datasets for all above threshold analyses used in this study.** Datasets are referenced in text from 1–80 and indicated is the Oncomine nomenclature for a study, the original publication reference and sample descriptions. (Information from www.oncomine.org , Compendia Bioscience, Ann Arbor, MI).
(DOCX)
