# Supplementary material for: Altered Expression of Two-Pore Domain Potassium (K2P) Channels in Cancer
Source: PLoS One. 2013 Oct 7;8(10):e74589. doi: 10.1371/journal.pone.0074589 (PMC3792113; doi:10.1371/journal.pone.0074589)
Supplement: File S1 — Contains Tables S1, S2, and S3. Oncomine datasets for all above threshold analyses used in this study. Datasets are referenced in text from 1–80 and indicated is the Oncomine nomenclature for a study, the original publication reference and sample descriptions. (Information from www.oncomine.org , Compendia Bioscience, Ann Arbor, MI). (DOCX) [file pone.0074589.s001.docx]

**Table S1: Oncomine dataset reference list**

| **Ref** | **Oncomine dataset** | **Publication** | **Tumour samples histology and staging** |
| --- | --- | --- | --- |
| 1 | Blaveri Bladder 2 | Blaveri E et al. (2005) Clin Cancer Res 11: 4044-4055. | 74 Transitional and 6 invasive squamous cell; stage Ta-4. 3 normal. |
| 2 | Sanchez-Carbayo Bladder 2 | Sanchez-Carbayo M et al. (2006) J Clin Oncol 24: 778-789. | 81 infiltrating and 28 superficial; staging not described. 48 normal. |
| 3 | Bredel Brain 2 | Bredel M et al. (2005) Cancer Res 65: 8679-8689. | 31 glioblastomas (including 2 gliosarcomas), 8 oligodendrogliomas (5 oligodendrogliomas, 3 anaplastic oligodendrogliomas), 6 anaplastic oligoastrocytomas, 5 astrocytic tumors; stage 1-3. 4 normal. |
| 4 | French Brain | French PJ et al. (2005) Cancer Res 65: 11335-11344. | 23 anaplastic oligodendroglioma, 4 anaplastic oligoastrocytoma; staging not described. 6 normal. |
| 5 | Lee Brain | Lee J et al. (2006) Cancer Cell 9: 391-403. | 72 brain and CNS; staging not described. 3 normal neural stem cell samples. 26 brain and CNS cancer cell lines |
| 6 | Murat Brain | Murat A et al. (2008) J Clin Oncol 26: 3015-3024. | 80 glioblastomas; staging not described. 4 normal. |
| 7 | Pomeroy Brain | Pomeroy SL et al. (2002) Nature 415: 436-442. | 81 malignant gliomas; stage 3-4. 4 normal cerebellum. |
| 8 | Rickman Brain | Rickman DS et al. (2001) Cancer Res 61: 6885-6891. | 45 astrocytomas; stage 1-4. 6 normal temporal lobe samples. |
| 9 | Sun Brain | Sun L et al. (2006) Cancer Cell 9: 287-300. | 157 brain and CNS; stages 2-4. 23 normal brain. |
| 10 | TCGA Brain | The Cancer Genome Atlas - Glioblastoma Gene Expression Data  <http://tcga-data.nci.nih.gov/tcga/> | 525 glioblastoma; staging not described. 10 normal brain. |
| 11 | Finak Breast | Finak G et al. (2008). Nat Med 14, 518-527. | 53 breast tumor stroma from 50 ductal and 3 lobular invasive carcinomas; stage 1-3. 6 normal breast stroma. |
| 12 | Ma Breast 4 | Ma X-J et al. (2009) Breast Cancer Res 11. | 20 ductal in situ and 18 invasive ductal breast carcinoma; stage 1-3. 28 normal breat. |
| 13 | Perou Breast | Perou CM, Sorlie T, Eisen MB, van de Rijn M, Jeffrey SS, et al. (2000) Molecular portraits of human breast tumours. Nature 406: 747-752. | 62 breast carcinoma; stage 1-3. 3 normal breast. |
| 14 | Radvanyi Breast | Radvanyi L et al. (2005) PNAS 102: 11005-11010. | 54 breast carcinoma; stage 1-3. 9 normal breast. |
| 15 | Sorlie Breast | Sørlie T et al. (2001). PNAS 98: 10869-10874. | 78 breast carcinoma and 3 fibroadenoma; staging not described. 4 normal breast. |
| 16 | Sorlie Breast 2 | Sørlie T et al. (2003) PNAS 100: 8418-8423. | 160 breast carcinoma and 3 fibroadenoma; stage . 4 normal breast. |
| 17 | TCGA Breast | The Cancer Genome Atlas - Invasive Breast Carcinoma Gene Expression Data  <http://tcga-data.nci.nih.gov/tcga/> | 532 invasive breast carcinoma and 3 paired metastatic; stage 61 paired normal breast. |
| 18 | Turashvili Breast | Turashvili G et al. (2007) BMC Cancer 7: 55. | 5 ductal and 5 lobular breast carcinoma; stage 1-3. 10 normal duct and 10 normal lobules. |
| 19 | Zhao Breast | Zhao H et al. (2004) Molecular Biology of the Cell 15: 2523-2536. | 61 breast carcinomas; stage 1-3. 5 normal breast. |
| 20 | Pyeon Multi-cancer | Pyeon D et al. (2007) Cancer Res 67: 4605-4619. | 42 head and neck cancers; stage 1-4. 14 head and neck normals.  20 cervical cancers; stage 1-4. 8 cervical normals. |
| 21 | Scotto Cervix 2 | Scotto L et al. (2008) Genes Chromosomes Cancer 47: 755-765. | 33 cervical carcinoma samples; stage ?. 24 normal cervix samples. 9 cervical carcinoma cell lines. |
| 22 | Zhai Cervix | Zhai Y et al. (2007) Cancer Res 67: 10163-10172. | 21 cervical squamous cell carcinoma and 7 high grade cervical squamous intraepithelial neoplasia; stage ?. 10 normal cervical squamous epithelia. |
| 23 | Hong Colorectal | Hong Y et al. (2010) Clin Exp Metastasis 27: 83-90. | 70 colorectal carcinoma; stage 1-3.12 normal colon. |
| 24 | Sabates-Bellver Colon | Sabates-Bellver J et al. (2007) Mol Cancer Res 5: 1263-1275. | 32 colorectal carcinomas; stage 0-1p (Paris classification). 32 matched normal. |
| 25 | Skrzypczak Colorectal 2 | Skrzypczak M et al. (2010) PLoS One 5: e13091. | 40 microdissected samples; 5 replicates each of 8 types of epithelial or mucosa cells from tumor or normal tissues. |
| 26 | TCGA colorectal | The Cancer Genome Atlas - Colon and Rectum Adenocarcinoma Gene Expression Data  <http://tcga-data.nci.nih.gov/tcga/> | 215 colorectal adenocarcinoma; high and low grade dysplasia. 22 paired normal colorectal tissue. |
| 27 | Cho Gastric | Cho JY et al. (2011) Clin Cancer Res 17: 1850-1857. | 65 gastric adenocarcinoma; stage 1-4. 6 gastrointestinal stromal tumor. 19 paired normal tissue and. were analyzed. |
| 28 | Derrico Gastric | D'Errico M et al. (2009) Eur J Cancer 45: 461-469. | 31 paired gastric carcinoma and adjacent normal gastric mucosa. 7 unmatched gastric carcinoma. Stage 2-3. |
| 29 | Wang Gastric | Wang Q et al. (2012) Med Oncol 29: 77-83. | 12 paired gastric carcinoma and normal gastric mucosa samples; stage 1-4. 3 normal gastric tissue. |
| 30 | Cromer Head-Neck | Cromer A et al. (2004) Oncogene 23: 2484-2498. | 34 head and neck squamous cell carcinoma; stage 1-4. 4 normal uvula samples. |
| 31 | Estilo Head-Neck | Estilo CL et al. (2009) BMC Cancer 9: 11. | 32 tongue squamous cell carcinoma; stage 1-4. 26 paired normal tongue samples. |
| 32 | Ginos Head-Neck | Ginos MA (2004) Cancer Res 64: 55-63. | 41 head and neck squamous cell carcinoma; stage 1-4. 13 normal buccal mucosa samples. |
| 33 | Pyeon Multi-cancer | Pyeon D et al. (2007) Cancer research 67, 4605-4619. | 42 head and neck cancers; stage 1-4. 14 head and neck normal.  20 cervical cancers; stage 1-4. 8 cervical normal. |
| 34 | Talbot Lung | Talbot SG et al. (2005) Cancer Res 65: 3063-3071. | 34 squamous cell lung carcinoma, 31 tongue squamous cell carcinoma; stages 1-4. 26 normal tongue, and 2 normal lung samples. |
| 35 | Ye Head-Neck | Ye H et al. (2008) BMC Genomics 9: 69. | 26 tongue squamous cell carcinoma samples; stage not described. 12 normal tongue squamous cell samples. |
| 36 | Beroukhim Renal | Beroukhim R et al. (2009) Cancer Res 69: 4674-4681. | 59 VHL-associated clear cell renal cell carcinoma samples; stage not described. 11 normal renal cortex samples. |
| 37 | Cutcliffe Renal | Cutcliffe C et al. (2005) Clin Cancer Res 11: 7986-7994. | 18 Wilms tumor samples, 14 clear cell sarcoma of the kidney samples; stage not described. 3 normal fetal kidney samples. |
| 38 | Gumz Renal | Gumz ML et al. (2007) Clin Cancer Res 13: 4740-4749. | 10 clear cell renal cell carcinoma samples; stage 1-4. 10 patient-matched normal tissue. |
| 39 | Jones Renal | Jones J et al. (2005) Clin Cancer Res 11: 5730-5739. | 32 clear cell renal cell carcinoma, 12 renal oncocytoma, 11 papillary renal cell carcinoma, 8 renal pelvis urothelial carcinoma, 6 chromophobe renal cell carcinoma; stages 1-4. 23 normal kidney samples. |
| 40 | Lenburg Renal | Lenburg ME et al. (2003) BMC Cancer 3. | 9 clear cell renal cell carcinoma; stage 1-3. 9 normal kidney samples. |
| 41 | Yusenko Renal | Yusenko MV et al. (2009) BMC Cancer 9: 152. | 26 conventional renal cell carcinomas, 19 papillary renal cell carcinomas, 4 chromophobe renal cell carcinomas, 4 Wilms tumors, 4 renal oncocytomas, 2 collecting duct carcinomas, 1 clear cell sarcoma of the kidney, 1 renal lipoma, 1 rhabdoid tumor of the kidney; stages not described. 3 normal adult kidney, and 2 normal fetal kidney samples. |
| 42 | Choi Leukemia | Choi YL et al. (2007) Oncogene 26: 1245-1255. | 22 acute adult t-cell leukemia/lymphoma, 19 chronic adult t-cell leukemia/lymphoma. 6 normal t-lymphocyte samples. |
| 43 | Haferlach Leukemia | Haferlach T et al. (2010) J Clin Oncol 28: 2529-2537. | 2,096 leukemia samples; stage C1-C18. Healthy bone marrow specimens or a variety of non-leukemia conditions. |
| 44 | Stegmair Leukemia | Stegmaier K et al. (2004) Nat Genet 36: 257-263. | 81 leukemia (60 of which were the acute promyelocytic leukemia cell line HL-60 and 12 of which were acute promyelocytic leukemia cell cultures) and 6 normal blood samples. AML and APL |
| 45 | Wurmbach Liver | Wurmbach E et al. (2007) Hepatology 45: 938-947. | 13 cirrhotic liver, 17 dysplastic liver, and 35 hepatocellular carcinoma; poor-well differentiated. 10 normal liver. |
| 46 | Beer Lung | Beer DG et al. (2002) Nat Med 8: 816-824. | 86 lung adenocarcinoma. 10 normal lung samples |
| 47 | Bhattaherjee Lung | Bhattacharjee A et al. (2001) PNAS 98: 13790-13795. | 139 lung adenocarcinoma, 21 squamous cell lung carcinoma, 20 lung carcinoid tumor, 6 small cell lung carcinoma; stages 1-3. 17 normal lung. |
| 48 | Garber Lung | Garber ME et al. (2001) PNAS 98: 13784-13789. | 67 lung carcinoma samples; various types stage 1-4. 6 normal lung. |
| 49 | Hou Lung | Hou J et al. (2010) PLoS One 5: 10312. | 91 non-small cell lung carcinoma; stage 1-4. 65 adjacent normal lung. |
| 50 | Landi Lung | Landi MT et al. (2008) PLoS One 3: 1651. | 107 lung adenocarcinoma (stage 1-2) and normal lung samples. |
| 51 | Stearman Lung | Stearman RS et al. (2005) The American Journal of Pathology 167: 1763-1775. | 10 invasive non-small cell lung adenocarcinoma; stage 1-3. 10 adjacent normal tissues. |
| 52 | Su Lung | Su LJ et al. (2007) BMC Genomics 8: 140. | 26 lung adenocarcinomas with paired adjacent normals, 1 large cell lung carcinoma with paired adjacent normal, 2 tissue mixtures, 2 commercial human normal lung tissues, 1 normal lung cell line, and 7 lung cancer cell lines. Stages not described. |
| 53 | Wachi Lung | Wachi S et al. (2005) Bioinformatics 21: 4205-4208. | 5 squamous cell lung carcinoma. 5 normal lung samples. |
| 54 | Brune Lymphoma | Brune V et al. (2008) J Exp Med 205: 2251-2268. | 42 malignant lymphoma samples, including 11 Hodgkin's lymphoma, 11 diffuse large B-cell lymphoma, 5 nodular lymphocyte predominant Hodgkin's lymphoma, 5 follicular lymphoma, 5 Burkitt's lymphoma, and 4 T-cell/histiocyte-rich large B-cell lymphoma samples were analyzed. 25 normal B-cell samples. |
| 55 | Storz Lymphoma | Storz MN et al. (2003) J Investig Dermatol 120: 865-870. | 19 lymphoma, 2 mycosis fungoides, 3 normal skin, and 3 normal tonsil samples. |
| 56 | Haqq Melanoma | Haqq C et al. (2005) PNAS 102: 6092-6097. | 25 melanoma, 9 non-neoplastic nevus, and 3 normal skin samples. |
| 57 | Riker Melanoma | Riker A et al. (2008) BMC Medical Genomics 1: 13. | 40 metastatic melanoma, 42 primary skin cancer, 4 normal skin, and 1 normal skin primary cell culture samples. |
| 58 | Talantov Melanoma | Talantov D et al. (2005) Clin Cancer Res 11: 7234-7242. | 45 cutaneous melanoma stage 1-4. 18 benign melanocytic skin nevus and 7 normal skin. |
| 59 | Zhan Myeloma 3 | Zhan F et al. (2007) Blood 109: 1692-1700. | 22 normal bone marrow, 44 monoclonal gammopathy of undetermined significance, and twelve 12 multiple myeloma samples. |
| 60 | Hao Oesophagus | Hao Y et al. (2006) Gastroenterology 131: 925-933. | 48 samples from 17 patients. 15 normal esophagus, 14 normal duodenum, 14 Barretts esophagus and 5 esophageal adenocarcinoma. |
| 61 | Kim Oesophagus | Kim SM et al. (2010) PLoS One 5: e15074. | 75 esophageal adenocarcinoma samples; stage 1-4, from 64 patients. 28 paired normal esophageal and 15 Barrett's esophagus samples. |
| 62 | Kimchi Oesophagus | Kimchi ET et al. (2005) Cancer Res 65: 3146-3154. | 24 paired samples of normal esophageal epithelium, Barretts metaplasia, and esophageal adenocarcinomas. |
| 63 | Wang Oesophagus | Wang S et al. (2006) Oncogene 25: 3346-3356. | 19 Barretts esophagus, 9 esophageal adenocarcinoma, and 24 normal esophagus samples. |
| 64 | Crabtree Uterus (Other) | Crabtree JS et al. (2009) Cancer Res 69: 6171-6178. | 23 matched uterine corpus leiomyoma and normal myometrium samples. |
| 65 | Giordana Adrenal (Other) | Giordano TJ et al. (2003) The American Journal of Pathology 162: 521-531. | 16 adrenal cortex carcinoma; stages low to high. 3 normal adrenal cortex samples. |
| 66 | Gordon Mesothelioma (Other) | Gordon GJ et al. (2005) The American Journal of Pathology 166: 1827-1840. | 44 mesothelioma (four of which are cell lines); stage 1-3. 5 normal pleural tissue, 4 normal lung and 1 normal mesothelium cell line samples. |
| 67 | Korkola Seminoma (Other) | Korkola JE et al. (2006) Cancer Res 66: 820-827. | 101 adult male germ cell tumor samples. 6 normal testis samples. |
| 68 | Nindl Skin (Other) | Nindl I et al. (2006) Mol Cancer 5: 30. | 15 total samples; 6 normal skin , 4 actinic keratosis and 5 squamous cell carcinoma. |
| 69 | Quade Uterus (Other) | Quade BJ et al. (2004) Genes Chromosomes Cancer 40: 97-108. | 20 smooth muscle neoplasms. 4 normal myometrium samples. |
| 70 | Skotheim Testis (Other) | Skotheim RI et al. (2005) Cancer Res 65: 5588-5598. | 23 testicular germ cell tumors; premalignant IGCN (n=3), seminoma (n=3), embryonal carcinoma (n=5), yolk sac tumor (n=4), choriocarcinoma (n=1), teratoma (n=4). 3 normal testicular parenchyma. 6 samples from 2 embryonal carcinoma cell lines. |
| 71 | Badea Pancreas | Badea L et al. (2008) Hepatogastroenterology 55: 2016-2027. | 39 pancreatic ductal adenocarcinoma. 39 normal pancreas. |
| 72 | Iacobuzio-Danahue Pancreas 2 | Iacobuzio-Donahue CA et al. (2003) The American Journal of Pathology 162: 1151-1162. | 14 pancreatic carcinoma cell lines, 17 primary pancreatic carcinoma samples of various histologies. 5 normal pancreas samples. |
| 73 | Logsdon Pancreas | Logsdon CD et al. (2003) Cancer Res 63: 2649-2657. | 17 pancreatic carcinoma; including 7 cell lines), 5 pancreatitis, and 5 normal pancreas samples. |
| 74 | Pei Pancreas | Pei H et al. (2009) Cancer Cell 16: 259-266. | 36 pancreatic carcinoma. 16 paired normal samples. |
| 75 | Segara Pancreas | Segara D et al. (2005) Clin Cancer Res 11: 3587-3596. | 11 pancreatic carcinoma samples; stage 1-4. 6 adjacent normal pancreas samples. |
| 76 | Arredouani Prostate | Arredouani MS et al. (2009) Clin Cancer Res 15: 5794-5802. | 13 prostate carcinoma samples. 8 normal prostate. not described |
| 77 | Grasso Prostate | Grasso CS et al. (2012) Nature 487: 239-243. | 35 metastatic prostate cancer, 59 localized prostate carcinoma; high grade. 28 benign prostate tissue. |
| 78 | Tomlins Prostate | Tomlins SA et al. (2007) Nat Genet 39: 41-51. | 101 prostate cancer cell types; benign, PIN, low-grade PCA, high-grade PCA, metastatic PCA) and normal adjacent tissue. |
| 79 | Vermbally Prostate | Varambally S et al. (2005) Cancer Cell 8: 393-406. | 6 metastatic prostate carcinoma samples and 7 primary prostate carcinoma samples. 6 normal prostate gland samples. |
| 80 | Detwiller Sarcoma | Detwiller KY et al. (2005) Cancer Res 65: 5881-5889. | 39 sarcoma and 15 normal tissue samples of various types. |

**Table S2: Twenty cancer types examined.**

| **Cancer** | **# threshold datasets** | **Meta-analysed** | |
| --- | --- | --- | --- |
| Bladder | 3 | X | |
| Brain and CNS | 8 | X | |
| Breast | 9 |  | |
| Cervical | 3 | X | |
| Colorectal | 4 | X | |
| Esophageal | 4 | X | |
| Gastrointestinal | 3 | X | |
| Head and Neck | 6 | X | |
| Kidney | 6 | X | |
| Leukemia | 3 | X | |
| Liver | 1 |  | |
| Lung | 8 | X | |
| Lymphoma | 2 |  | |
| Melanoma | 3 | X | |
| Myeloma | 1 |  | |
| Other | 7 |  | |
| Ovarian | 0 |  | |
| Pancreatic | 5 | X | |
| Prostate | 4 | X | |
| Sarcoma | 1 |  |  |

List of 20 cancer types selected for initial analysis. Number of above threshold datasets for KCNK gene expression is indicated. Cancer types which had sufficient number of studies (n ≥ 5) for inclusion in comparative meta-analysis is also indicated.

**Table S3: Above-threshold analyses of KCNK expression in cancer subtypes excluded from meta-analysis.**

| **Gene** | **Cancer** | **Subtype** | **Above threshold analyses** | | | | |
| --- | --- | --- | --- | --- | --- | --- | --- |
|  |  |  |  | **p-value** | **Fold change** | **%** | **Ref** |
| KCNK1 | Bladder | Infiltrating | ↑ | 1.05E-10 | 2.283 | 1 | 2 |
|  |  |  | ↑ | 6.67E-08 | 2.701 | 7 | 1 |
|  |  | Superficial | ↑ | 1.12E-16 | 5.285 | 3 | 2 |
|  |  |  | ↑ | 2.79E-08 | 2.385 | 1 | 1 |
|  | Brain | Astrocytoma | ↓ | 3.75E-08 | -4.168 | 4 | 9 |
|  |  |  | ↓ | 8.09E-08 | -6.64 | 1 | 8 |
|  |  | Medulloblastoma | ↓ | 2.60E-08 | -14.207 | 2 | 7 |
|  |  |  | ↓ | 5.48E-05 | -10.211 | 2 | 7 |
|  |  | Oligodendrioglioma | ↓ | 5.42E-16 | -4.813 | 1 | 9 |
|  |  |  | ↓ | 2.00E-03 | -3.963 | 9 | 4 |
|  | Esophageal | Barrett's | ↑ | 1.60E-02 | 2.352 | 7 | 62 |
|  | Head and Neck | Tongue squamous cell | ↑ | 2.98E-10 | 2.277 | 3 | 34 |
|  |  |  | ↑ | 9.00E-05 | 2.784 | 3 | 35 |
|  |  |  | ↑ | 3.35E-06 | 2.88 | 10 | 31 |
|  | Melanoma | Melanoma | ↓ | 7.58E-04 | -2.769 | 4 | 56 |
|  |  |  | ↓ | 1.55E-04 | -5.725 | 3 | 57 |
| KCNK2 | Gastrointestinal | Adenocarcinoma | ↓ | 6.43E-05 | -5.469 | 2 | 28 |
|  | Head and Neck | Squamous cell | ↓ | 2.24E-05 | -3.693 | 2 | 30 |
| KCNK3 | Bladder | Inflitrating | ↓ | 6.57E-13 | -3.045 | 5 | 1 |
|  |  | Superficial | ↓ | 5.79E-11 | -4.046 | 6 | 1 |
|  | Brain | Astrocytoma | ↓ | 3.00E-03 | -2.156 | 9 | 9 |
|  | Colorectal | Colon adenoma | ↓ | 2.37E-04 | -2.493 | 10 | 24 |
|  | Leukemia | Chronic Lymphocytic | ↑ | 4.00E-03 | 2.681 | 5 | 42 |
| KCNK5 | Esophageal | Adenocarcioma | ↑ | 5.17E-12 | 2.077 | 4 | 61 |
|  |  |  | ↑ | 2.31E-04 | 2.481 | 2 | 62 |
|  |  |  | ↓ | 6.98E-04 | -2.281 | 8 | 63 |
|  |  | Barrett's | ↑ | 1.60E-11 | 5.57 | 1 | 61 |
|  |  |  | ↑ | 1.50E-02 | 2.019 | 7 | 62 |
|  | Kidney | Clear cell | ↓ | 1.07E-04 | -3.693 | 3 | 40 |
|  |  |  | ↓ | 1.67E-04 | -2.409 | 9 | 38 |
|  | Leukemia | Acute Myeloid | ↓ | 1.30E-02 | 2.172 | 8 | 44 |
|  |  | Chronic Lymphocytic | ↓ | 4.97E-39 | -2.039 | 5 | 43 |
|  | Lung | Adenocarcinoma | ↑ | 4.27E-06 | 2.145 | 3 | 52 |
|  |  | Squamous cell | ↓ | 9.14E-14 | -2.192 | 6 | 49 |
|  | Melanoma | Melanoma | ↓ | 5.02E-10 | -6.576 | 2 | 58 |
|  |  |  | ↓ | 4.00E-03 | -2.869 | 10 | 57 |
| KCNK6 | Breast | Invasive | ↑ | 5.44E-04 | 3.237 | 1 | 14 |
|  | Esophageal | Adenocarcinoma | ↓ | 2.20E-12 | -2.223 | 8 | 61 |
|  | Melanoma | Melanoma | ↓ | 2.01E-04 | -2.209 | 3 | 57 |
|  |  |  |  |  |  |  |  |
| KCNK7 | Esophageal | Adenocarcinoma | ↓ | 2.40E-17 | -2.681 | 4 | 61 |
|  |  |  | ↓ | 3.74E-04 | -9.38 | 2 | 62 |
|  |  | Barrett's | ↓ | 1.12E-10 | -2.608 | 5 | 61 |
|  |  |  | ↓ | 3.00E-03 | -2.586 | 3 | 60 |
|  | Head and Neck | Squamous cell | ↓ | 2.80E-11 | -4.753 | 4 | 32 |
|  | Melanoma | Melanoma | ↓ | 7.33E-09 | -64.334 | 1 | 57 |
| KCNK10 | Breast | Lobular | ↓ | 3.50E-02 | -3.576 | 5 | 14 |
|  | Colorectal | Adenoma | ↓ | 3.61E-11 | -2.758 | 1 | 25 |
|  |  | Carcinoma | ↓ | 5.65E-10 | -3.37 | 5 | 23 |
|  |  |  | ↓ | 7.55E-08 | -2.155 | 3 | 25 |
|  |  |  | ↓ | 7.17E-07 | -2.41 | 5 | 25 |
|  | Kidney | Clear cell | ↓ | 2.17E-10 | -4.752 | 1 | 38 |
|  |  |  | ↓ | 1.11E-06 | -2.748 | 7 | 36 |
|  |  |  | ↓ | 1.19E-06 | -2.783 | 5 | 36 |
|  | Leukemia | Acute Myeloid | ↓ | 2.00E-02 | 2.615 | 10 | 44 |
| KCNK12 | Brain | Astrocytoma | ↓ | 8.77E-08 | -4.126 | 5 | 9 |
|  |  |  | ↓ | 3.00E-03 | -3.054 | 10 | 9 |
|  |  | Glioblastoma | ↓ | 1.81E-23 | -7.969 | 2 | 9 |
|  |  |  | ↓ | 3.32E-09 | -4.925 | 8 | 10 |
|  | Head and Neck | Squamous cell | ↓ | 2.99E-07 | -7.542 | 9 | 32 |
|  | Leukaemia | Acute Lymphocytic | ↑ | 5.75E-75 | 3.532 | 1 | 43 |
|  |  |  | ↑ | 4.96E-34 | 3.301 | 3 | 43 |
|  |  |  | ↑ | 5.99E-33 | 8.691 | 2 | 43 |
|  | Lung | Adenocarcinoma | ↑ | 9.84E-06 | 2.803 | 4 | 52 |

**Table S3: Above-threshold analyses of KCNK expression in cancer subtypes excluded from meta-analysis.**

The above threshold data for KCNK expression in cancer subtypes in which insufficient numbers of studies were available to enable inclusion in meta-analysis (n ≤ 4) is shown. Data are grouped by gene name and divided into each cancer type and subtypes within that cancer. The p-value, fold change and gene rank percentile (%) for data which scored above threshold values (p-value < 0.05, fold change > 2 and gene rank percentile < 10 %) are shown. Overexpression ↑ and underexpression ↓ are indicated.
